# Human Detection Based on the Generation of a Background Image and Fuzzy System by Using a Thermal Camera

**DOI:** 10.3390/s16040453

**Published:** 2016-03-30

**Authors:** Eun Som Jeon, Jong Hyun Kim, Hyung Gil Hong, Ganbayar Batchuluun, Kang Ryoung Park

**Affiliations:** Division of Electronics and Electrical Engineering, Dongguk University, 30 Pildong-ro 1-gil, Jung-gu, Seoul 100-715, Korea; jeunsom@dgu.edu (E.S.J.); zzingae@dongguk.edu (J.H.K.); hell@dongguk.edu (H.G.H.); ganabata87@gmail.com (G.B.)

**Keywords:** human detection, thermal camera image, generation of background image, fuzzy system

## Abstract

Recently, human detection has been used in various applications. Although visible light cameras are usually employed for this purpose, human detection based on visible light cameras has limitations due to darkness, shadows, sunlight, *etc*. An approach using a thermal (far infrared light) camera has been studied as an alternative for human detection, however, the performance of human detection by thermal cameras is degraded in case of low temperature differences between humans and background. To overcome these drawbacks, we propose a new method for human detection by using thermal camera images. The main contribution of our research is that the thresholds for creating the binarized difference image between the input and background (reference) images can be adaptively determined based on fuzzy systems by using the information derived from the background image and difference values between background and input image. By using our method, human area can be correctly detected irrespective of the various conditions of input and background (reference) images. For the performance evaluation of the proposed method, experiments were performed with the 15 datasets captured under different weather and light conditions. In addition, the experiments with an open database were also performed. The experimental results confirm that the proposed method can robustly detect human shapes in various environments.

## 1. Introduction

With the recent development of computer vision and pattern recognition technologies, human detection has been used in various applications, including intelligent surveillance systems [1,2,3,4,5,6,7,8,9]. Because of its various advantages of being less affected by poor conditions like illumination changes, low light, and fog, *etc.*, human detection by thermal cameras has been highlighted. The gray level value of an object in a thermal image is determined by the temperature of the object. Generally, humans in an image are warmer than the background, so the gray level of a human’s area is usually higher than that of the surrounding environment. However, the properties of these areas can be affected by the temperature or environmental conditions. The condition that a human area is brighter than the surrounding areas in a thermal image is typically satisfied during night and winter, but in summer, the condition is changed, and the brightness of a human image is darker than the background during summer or on a hot day. These factors can affect the accuracy of detecting human areas in thermal images [9,10,11,12,13,14,15,16,17,18,19] and make it difficult to distinguish human areas from the background in the image.

In order to overcome these drawbacks and to extend the applications of human tracking and behavioral recognition, various researches have recently focused on detecting human areas. There are several previous studies related to human detection in thermal camera images. These can be divided into two categories: those without background models [4,5,10,11,12,13,14,15,16,17,18,19,20], and those with background models [21,22,23,24,25,26,27,28,29,30,31,32,33,34,35,36]. In the former category, some methods have employed features based on the histogram of the oriented gradient (HOG) [4,5,10,11,12,13,14,15] with a support vector machine (SVM) [13,14,15], the soft-label [16], and the edge features with an adaptive boosting method (Adaboost) [17]. Fuzzy systems are used to classify human areas without using background information [18,19,20]. The advantage of these methods is that procedures for constructing backgrounds are not required, but they do however require training procedures for extracting or obtaining a pre-defined template, as well as various types of scales for the detection of different sizes of humans. In addition, various conditions from images captured at different times and from different views can affect the accuracy of the results. They also require a significant amount of processing time to detect humans because of the need to scan the entire region of the image.

Because of these drawbacks, human detection methods with background models have been employed as an alternative. The Gaussian approach [21,22,23], expectation minimization [24,25], texture change [26], and statistical methods [22,27,28,29,30,31,32] are used to create a background image. In addition, image averaging [32,33,34,35] or running average methods [35,36] can be used for background modeling. After extracting the background information, contour saliency maps (CSM) [21,22,23], template matching with CSM [32], and shape- and appearance-based features obtained using principal component analysis (PCA) [24,25] are used for human detection. Spatiotemporal texture vectors [26] can also be applied. In previous studies [34,35], fuzzy-based methods for background subtraction have been employed. The advantage of these methods is that they can be applied to multiple conditions of images that have various object sizes. These methods can also be applied to various environmental conditions such as snow, rain, sunlight, night, and daytime. However, their performance is influenced by the similarity between the background and the object because these performances are based on a background subtraction method. That is, they did not consider the cases where the background is similar to the object in the image. In addition, if there are motionless people located in the same positions in all the frames, these people can be factors for generating erroneous backgrounds, and degradation of performance for human detection can therefore occur because of these erroneous backgrounds.

To overcome these drawbacks, we present herein a new approach to detect human areas in a thermal image under varying environmental conditions. The proposed research is novel in the following four respects:

- First, the threshold for background subtraction is adaptively determined based on a fuzzy system. This system uses the information derived from the background image and difference values between the background and input image.
- Second, the problem of two or more than two people being in the similar place with occlusion is solved by our method. Based on four conditions (the width, height, size, and ratio of height to width), the candidate region is separated into two parts. In addition, if the width or height of the detected box is larger than a threshold, our algorithm also checks whether there exist two or more than two histogram values which are lower than the threshold. If so, the candidate region is horizontally or vertically divided into three or more than three regions at the positions of the histogram values.
- Third, for human confirmation, the separated regions are verified based on the size and the distance between two or more regions in close proximity to one another. If a region is small and there is another small region nearby, these two regions are merged as an exact human region.
- Fourth, our method is confirmed to robustly detect human areas in various environments through intensive experiments with 15 sets of data (captured under different weather and light conditions) and an open database.

The main contribution and advantage of our method are that the thresholds for creating the binarized difference image between the input and background images are determined adaptively based on fuzzy systems by using the information derived from the background image and difference values between background and input image. By using our method, human areas can be correctly detected, irrespective of the various conditions of input and background images, which can be a crucial requirement for intelligent surveillance system applications. Our work is suitable only for the case of intelligent surveillance using static cameras, therefore, we do not consider cases with dynamic background such as advanced driver assistance systems in our research. In previous research [37], the authors proposed a method for detecting human areas, but the above four novel points of our research are different from the previous research [37].

The remainder of this article is organized as follows: we provide an overview of the proposed system and an algorithm for human detection in Section 2. We present the experimental results and analysis in Section 3. Finally, the conclusions are discussed in Section 4.

## 2. Proposed Method

### 2.1. Overall Procedure of Proposed Method

**Figure 1:**
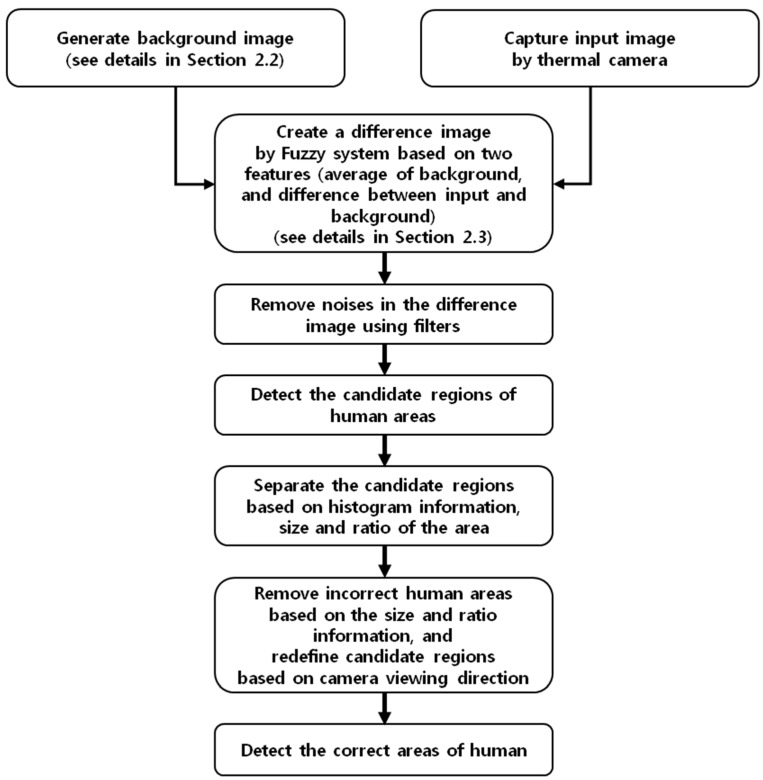
Overall procedure of the proposed method.

An overview of the proposed method is presented in Figure 1. We propose a three step system for detecting humans in a thermal image: generation of a background image (model); obtaining a difference image based on fuzzy system with the background and input image; and detection of humans in the difference image. In this paper, the image obtained in the sub-bands of medium-wave IR (MWIR, 3–8 μm) and long-wave IR (LWIR, 8–15 μm) is called a thermal image [38].

First, a background image is generated. An image is created by filtering, and non-background areas are removed. In addition, a correct background image is created (see the details in Section 2.2). Then, a (pixel) difference image is obtained from the background and input images. The threshold for extracting a candidate area is adaptively determined using a fuzzy system which uses the brightness feature of the generated background and input image (see the details in Section 2.3). The third step is to detect human areas. Incorrect human areas are removed by size filtering and morphological operations. Based on a vertical and horizontal histogram and the size and ratio of the area, the candidate area is separated. After removing incorrect human areas based on the size and ratio of the candidate area, further procedures for detecting correct human area are performed. The remaining areas are merged adaptively based on the distance between the objects and camera viewing direction (see the details in Section 2.4). Finally, the correct human areas are obtained.

### 2.2. Generating a Background Image

Previous research used background subtraction methods with background modeling to detect human areas [21,22,23,24,25,26,27,28,29,30,31,32,33,34,35,36]. In order to detect human areas by the background subtraction method, generating a correct background image is necessarily required. Statistical methods [22,27,28,29,30,31,32], temporal averaging [31,32,33,34,35], and running average-based methods [36,38] are used for generating background images. However, there are some ghost shadows in a generated background image, which come about as the result of temporal averaging methods. Moreover, the research for making background images does not implement further procedures for considering motionless people in an image. If there is a motionless human in all frames which are used for creating a background image, erroneous background images can be generated.

In order to overcome this drawback, Dai *et al.* proposed a method to create a background image by using multiple images obtained from two other sequences [24]. However, the intensity of the created image from the procedure of making a background image is quite different from that of the input image. For instance, if a sequence obtained at daytime and other sequence obtained at night are used for creating a background by an averaging method, the generated background image has the average brightness of these two sequences, is the intensity of which is much different compared to the input image. Therefore, incorrect background images can be created and detection errors can occur.

**Figure 2:**
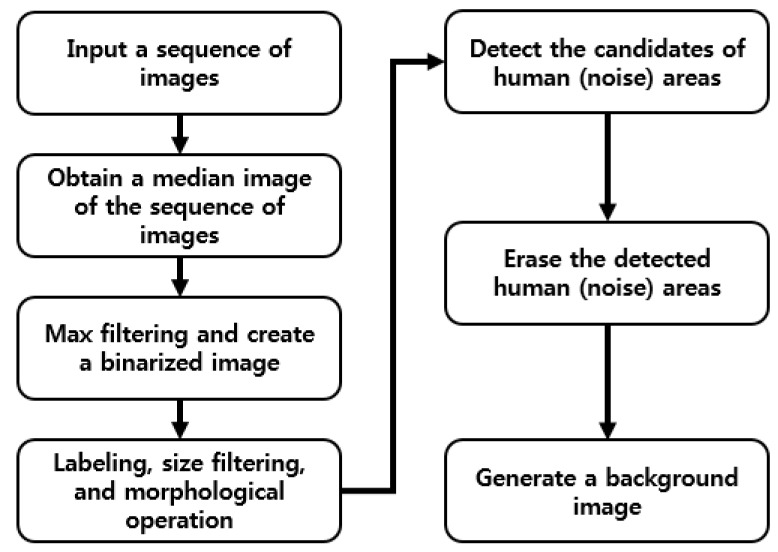
Flow chart of generating a background image (model).

**Figure 3:**
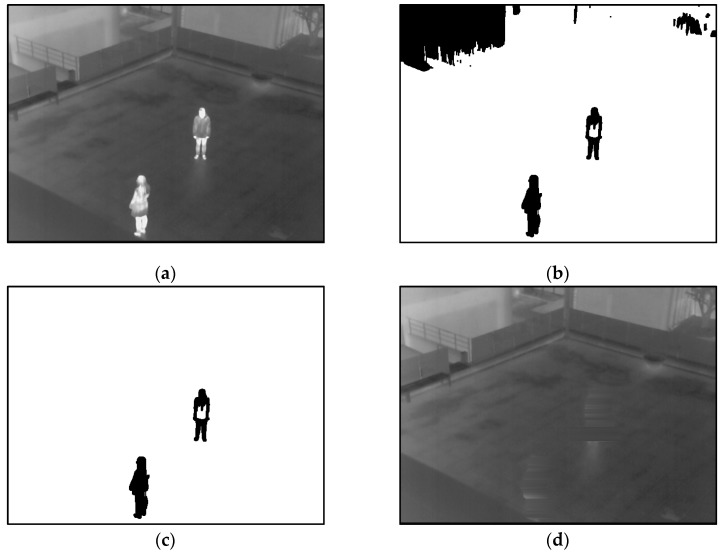
Examples of generating the background image from database I: (**a**) preliminary background image obtained by median value from the sequence of images; (**b**) extracted candidate human area by binarization; (**c**) extracted human areas by labeling, size filtering and a morphological operation; and (**d**) the final background image.

Therefore, we propose a method for creating a correct background image to overcome these problems. Our method of creating a correct background image is referred to previous research [37]. A flow chart of the proposed method is presented in Figure 2. To begin, a background image is generated by using training images. To solve the problem of ghost shadows in a background image, the median values of pixels from successive multiple frames (from 10 to 70 frames) in a sequence are used [22,27,28,29,30,31,32]. By using the median values, a median image, which corresponds to a preliminary background image, is created, as illustrated in Figure 3a. However, the motionless humans in all frames can cause the incorrect inclusion of non-background areas in the created image. Therefore, further procedures are performed to generate a correct background image. A 3 × 3 pixels max filter is applied to the created median image to enhance the human area compared to background area. In general, the gray level of a person in a thermal image is higher than that of background. Therefore, by max filtering, human area is shown to be more evident than the background. Based on the average and standard deviation value of the background image, a binary image which shows the candidate non-background area (human area) is created by Equation (3) [37,39]:

(1) $$\mu =\;\frac{\sum_{i=1}^{M}\sum_{j=1}^{N}I_{med}\left(i,j\right)}{M\times N}$$

(2) $$\sigma =\;\sqrt{\frac{\sum_{i=1}^{M}\sum_{j=1}^{N}{\left(I_{med}\left(i,j\right)-\mu \right)}^{2}}{M\times N-1}}$$

(3) $$B\left(i,j\right)=\{\;\begin{array}{c} 0\;if\;((I_{med}\left(i,j\right)<\mu -P\times \sigma )\;and\;(\mu >Q))\;or\\ \;((I_{med}\left(i,j\right)>\mu +P\times \sigma )\;and\;\left(\mu \leq Q\right))\;\\ 1\;otherwise \end{array}$$

where: *I_med_*(*i*, *j*) is the gray level value at the position (*i*, *j*) of a created median image; *M* and *N* are the width and height of the image, respectively; *μ* is the average and *σ* is standard deviation value of the median image; *B*(*i*, *j*) is a binary image, which presents candidate non-background area (human area); and *P* and *Q* are the optimal parameters, which are determined experimentally with the images (which were not used for all the experiments of performance measurements shown in Section 3).

With these images, the ground-truth areas of human were manually depicted. In addition, according to various *P* and *Q*, the human areas could be automatically detected based on the Equation (3). With the ground-truth and automatically detected areas, we can calculate the PPV, Sensitivity, and F1-score of the Equations (17)–(19). Optimal *P* and *Q* were determined, with which the highest PPV, Sensitivity, and F-score of human detection were obtained. The selected *P* and *Q* are 1.5 and 120.4, respectively. In addition, the same values of *P* and *Q* were used for all the experiments in Section 3.

In general, human areas are much smaller than the background area. Therefore, the average value of the median image (*μ* of the Equation (1)) determines the equation that should be applied for binarization. After binarization, the candidates of human areas are detected as shown in Figure 3b. In order to extract exact human areas to be erased, a component labeling and a morphological operation are applied to the binarized image. Through the component labeling, the pixel positions of isolated candidate area can be located [40]. Then, morphological operations including dilation and erosion on the candidate area can reduce the small-sized noises and combine the incorrectly separated regions [40]. In addition, component labeling and size filtering are performed to remove a great number of small or large areas, which are not regarded as human areas. Because the pixel positions of isolated candidate areas can be located through component labeling, the pixel number of the candidate area can be counted [40]. Based on the pixel number, small or large areas (which is difficult to be regarded as human area) can be removed (size filtering). The candidates of human areas extracted are shown in Figure 3c. These areas should be erased to generate a correct background image.

In order to erase these areas, we propose as erasing method with a detailed explanation. Linear interpolation is the main idea of this method [37]. If there is a candidate human area, the leftmost and rightmost positions of the candidate area for every row are determined as the X_1_ and X_2_ positions, respectively. Those positions smaller than X_1_ and larger than X_2_ are determined as the pixel positions (X_a_ and X_b_) of the background region, which implies nonhuman regions. After extracting X_a_ and X_b_ positions, the candidate human area is erased by linear interpolation with the pixel value of these positions based on the Equation (4). This procedure is performed iteratively for the entire image:

(4) $$Y=\;\frac{Y_{b}-Y_{a}}{X_{b}-X_{a}}\left(X-X_{a}\right)+Y_{a}$$

where X_a_ and X_b_ respectively represent the x positions of the background region; Y_a_ and Y_b_ are the pixel values of X_a_ and X_b_, respectively; X is an intermediate x position between X_a_ and X_b_; Y is the calculated pixel value of X by linear interpolation. After performing this erasing method, the human candidate area and its surrounding area can be replaced to near pixel values. That is, the human area that is a cause of the generation of erroneous background images can be removed.

Finally, a correct background image is created. Although motionless humans are located at the same position in all of the frames, all non-background areas (human areas) are erased and a correct image is generated. In this study, we used fifteen kinds of databases. More detailed experimental results with these databases are presented in Section 3.1. One example of the generated background image is provided in Figure 3d, where there are no human areas in the final background image.

**Figure 4:**
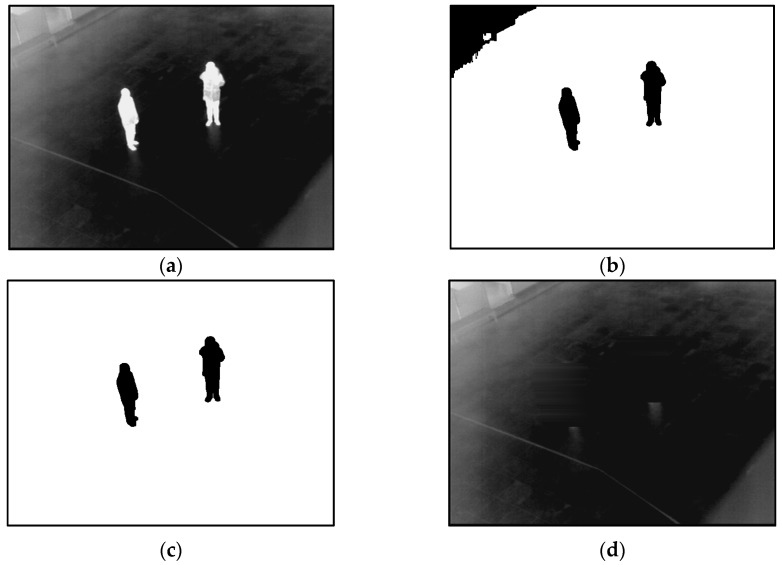
The first example for generating a background image from database III: (**a**) preliminary background image obtained by median value from the sequence of images; (**b**) extracted candidate human area by binarization; (**c**) extracted human areas by labeling, size filtering and a morphological operation; and (**d**) the final background image.

**Figure 5:**
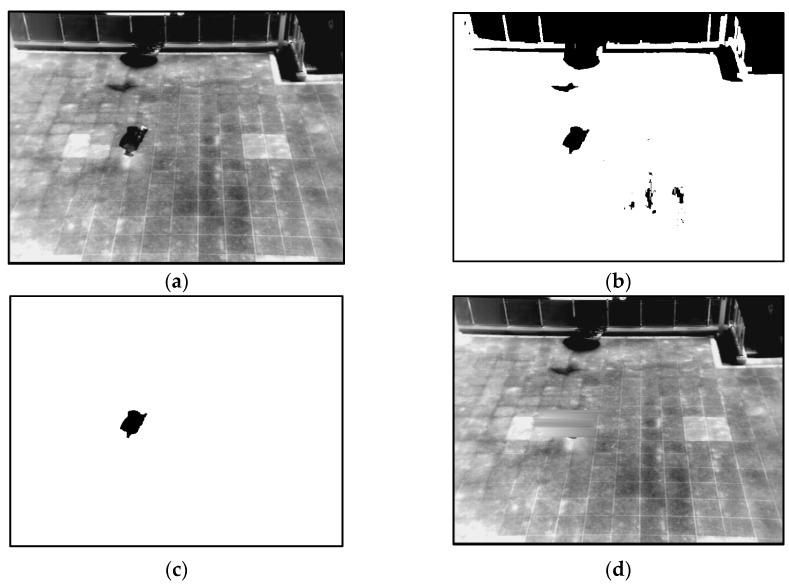
The second example for obtaining a background image from database VIII: (**a**) preliminary background image obtained by median value from the sequence of images; (**b**) extracted candidate human area by binarization; (**c**) extracted human areas by labeling, size filtering and a morphological operation; and (**d**) the final background image.

Two more examples of the generated background images are presented in Figure 4 and Figure 5. As illustrated in Figure 4 and Figure 5, a correct background image, which does not include human areas, is generated. That is, the proposed method can create correct background images for the procedure of human detection.

### 2.3. Generating a Difference Image Based on the Fuzzy System Given Background and Input Image

In the proposed method, in order to extract human candidate areas, a background subtraction method is used. First, to reduce noises which are usually generated in thermal image, 3 × 3 median filtering is applied to a generated background image before subtraction. Further, 3 × 3 median and average filtering is applied to an input image for reducing the noises. By using the difference between the background and input images, a binary image which presents candidate human areas is created. To create a difference image, the optimal threshold is required to cover the brightness variation of an image caused by various environmental conditions. In order to determine threshold adaptively, fuzzy system is used for the proposed method (see the details in Section 2.3.1).

#### 2.3.1. Definition of the Membership Function

**Figure 6:**
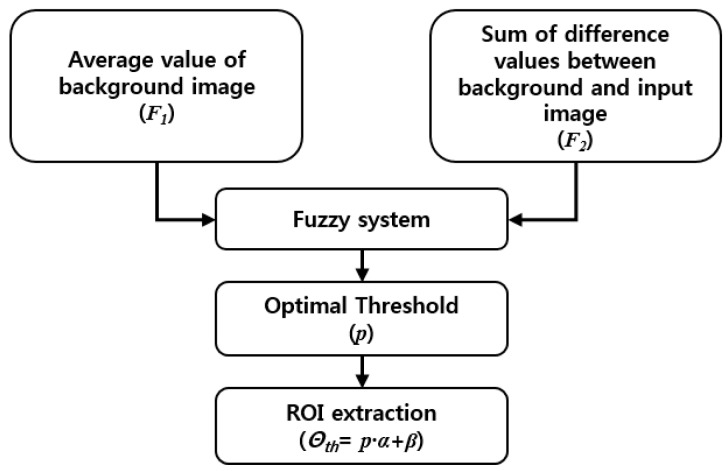
Fuzzy system for the proposed method to extract adaptive threshold for ROI extraction.

A fuzzy system for the proposed method is illustrated in Figure 6. The characteristics of human areas in thermal images are changed because of temperature and environmental factors. For example, in general, the intensity of humans in thermal images captured at night or during winter is much higher than that of the background. However, if the image is captured at daytime or much high temperature conditions, these intensity conditions are reversed. To consider these conditions, we use two kinds of features for the fuzzy system to extract the correct threshold for obtaining a human candidate area by background subtraction. The average value of the generated background image (*F*_1_ of the Equation (5)) and the sum of the difference values between the background and input images (*F*_2_ of the Equation (7)) are used as two input features. First, the average value of the generated background image is simply obtained using Equation (5):

(5) $$F_{1}=\;\frac{\sum_{i=1}^{M}\sum_{j=1}^{N}B\left(i,j\right)}{MXN}$$

where *B*(*i*, *j*) is the gray level value at the position (*i*, *j*) of a generated background image; *M* and *N* are the width and height of the image, respectively; and *F*_1_ is the average value of the generated background image.

To determine the condition stating whether intensity of human is higher than that of background or not, Equations (6) and (7) are employed. Based on Equations (6) and (7), the sum of the difference values between the background and input images is obtained:

(6) $$D_{t}\left(i,j\right)=\{\begin{array}{c} I_{t}\left(i,j\right)-B\left(i,j\right)\;if\;\left|I_{t}\left(i,j\right)-B\left(i,j\right)\right|>T)\\ \;0\;otherwise\; \end{array}$$

(7) $$F_{2}=\overset{M}{\underset{i=1}{\sum }}\overset{N}{\underset{j=1}{\sum }}D_{t}\left(i,j\right)$$

where: *B*(*i*, *j*) and *I_t_*(*i*, *j*) are the gray level value at the position (*i*, *j*) of a generated background and input image, respectively; and *T* is a threshold, which was experimentally determined with the images (which were not used for all the experiments of performance measurements shown in Section 3). With these images, according to various *T*, the pixels of candidate human areas could be distinguished in the image (*D_t_* (*i*, *j*)) based on Equation (6). By human observation on these generated images according to various *T*, optimal *T* (80) was determined, with which the candidate human areas could be most distinctive in the image. In addition, the same value of *T* was used for all the experiments in Section 3. *D_t_* (*i*, *j*) is determined by the absolute difference value between the background and input images. If the absolute difference value is larger than *T*, the position (*i*, *j*) is determined as the pixel of candidate area. In the Equation (7), *M* and *N* are the width and height of the image, respectively. *F*_2_ is the sum of the difference values between the background and input images. If *F*_2_ is higher than 0, the intensity of a human is higher than that of the background; otherwise, the intensity of a human is lower than that of background. The term *t* indicates the frame number of the input image in the sequence.

**Figure 7:**
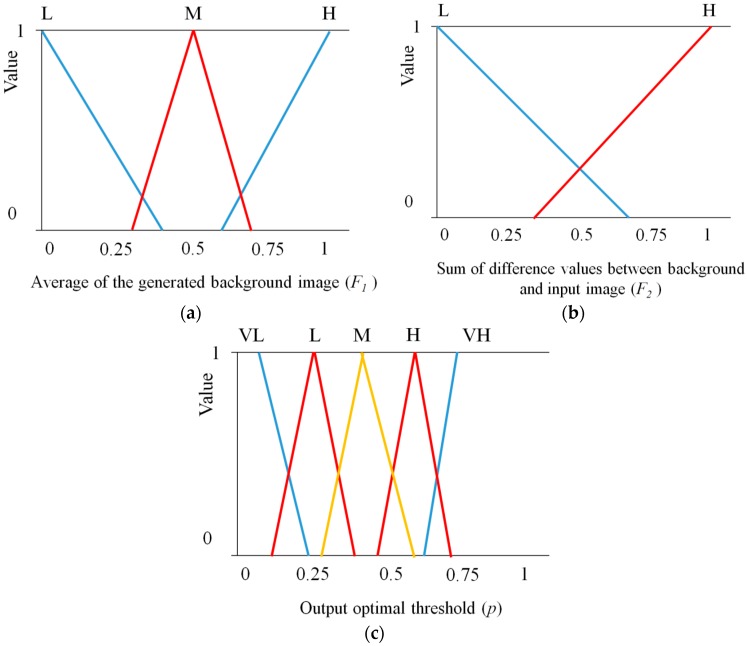
Membership functions for fuzzy system to extract adaptive threshold for ROI extraction: (**a**) average value of the background image; (**b**) sum of difference values between background and input image; and (**c**) obtaining the output optimal threshold.

For determining an effective threshold for obtaining regions of interest (ROI) which represent a human candidate area, the brightness of a background image is the main concern for the background subtraction technique. If the brightness difference between a human and the background is too small and the threshold for subtraction is too large, it is hard to extract a human area. On the other hand, if the brightness difference is too large and the threshold for subtraction is too small, it is also difficult to define a human area because other neighboring areas can be determined as human areas. Therefore, in order to represent membership functions for the brightness of background images, we use three membership functions for low (L), medium (M), and high (H), as shown in Figure 7a. 

To distribute the intensity conditions of humans compared to the background, the sum of the difference values is used with two membership functions for low (L) and high (H), as shown in Figure 7b. For an output optimal threshold which is used to ROI extraction, five membership functions are used. There are five types of linguistic values; very low (VL), low (L), medium (M), high (H), very high (VH), as illustrated in Figure 7c. An adaptive threshold to be used in the ROI extraction procedure is calculated with the output optimal threshold of the fuzzy system (see the details in Section 2.3.3). That is, the output threshold of the fuzzy system determines the human candidate area (see the details in Section 2.3.4). The linear (triangular) function is used because it has been widely adopted in fuzzy systems considering the problem complexity and its fast processing speed [41,42,43]. Like conventional researches using fuzzy system [41,42,43,44,45,46], the gradient and y-intercept of each linear function are manually determined based on the experience of human developer. Equations (8)–(10) show the mathematical definitions of Figure 7a–c, respectively:

(8) $$y=\{\begin{array}{c} -2.5x+1\;\left(0\leq x\leq 0.4\right)\;:\mathrm{Low}\\ \;5x-1.5\;\left(0.3\leq x\leq 0.5\right)\;:\mathrm{Medium}\\ -5x+3.5\;\left(0.5\leq x\leq 0.7\right)\;:\mathrm{Medium}\\ 2.5x-1.5\;\left(0.6\leq x\leq 1\right)\;:\mathrm{High} \end{array}$$

(9) $$y=\{\begin{array}{c} \left(-\frac{20}{13}\right)x+1\;\left(0\leq x\leq 0.65\right)\;:\mathrm{Low}\\ \left(\frac{20}{13}\right)x-\frac{7}{13}\;\left(0.35\leq x\leq 1\right)\;:\mathrm{High}\; \end{array}$$

(10) $$y=\{\begin{array}{c} 1\;\left(0\leq x\leq 0.1\right)\;:\text{Very Low}\\ -10x+2\;\left(0.1\leq x\leq 0.2\right)\;:\text{Very Low}\\ \;10x-1.5\;\left(0.15\leq x\leq 0.25\right)\;:\text{Low}\\ -10x+3.5\;\left(0.25\leq x\leq 0.35\right)\;:\text{Low}\\ 10x-3\;\left(0.3\leq x\leq 0.4\right)\;:\mathrm{Medium}\\ -10x+5\;\left(0.4\leq x\leq 0.5\right)\;:\text{Medium}\\ 10x-4.5\;\left(0.45\leq x\leq 0.55\right)\;:\mathrm{High}\\ -10x+6.5\;\left(0.55\leq x\leq 0.65\right)\;:\text{High}\\ 10x-6\;\left(0.6\leq x\leq 0.7\right)\;:\text{Very High}\\ 1\;\left(0.7\leq x\leq 1\right)\;:\text{Very High} \end{array}$$

#### 2.3.2. Fuzzy Rules with Considering the Characteristics of Background and Input Images

As described in Table 1, if the average value of the background image (*F*_1_) and the sum of the difference values between the background and input images (*F*_2_) is low (L) and high (H), respectively, the possibility of difference between the background and input images is very high (VH). Therefore, the output threshold (*p)* is determined with a large value. For high *F*_1_ and high *F*_2_, the possibility of difference between background and input image is very low (VL). That is, in this case, the intensity of a human is very similar to that of the background, and it has high pixel value. Based on these fuzzy rules, the output threshold (*p*) is determined.

#### 2.3.3. Decision of the Optimal Threshold Using Defuzzification

**Figure 8:**
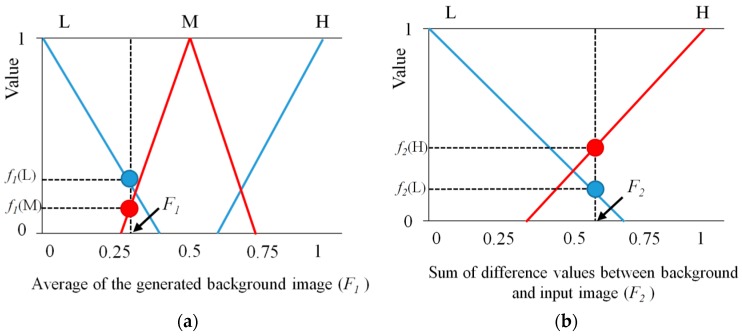
The first example for output of membership functions for fuzzy system: outputs by (**a**) *F*_1_ and (**b**) *F*_2_.

As illustrated in Figure 8, based on the *F*_1_ and *F*_2_, four output values (*f*_1_(L) and *f*_1_(M) for *F*_1_, and *f*_2_(L) and *f*_2_(H) for *F*_2_) are calculated. For example, in the first instance, we assume that *f*_1_(L), *f*_1_(M), *f*_2_(L), and *f*_2_(H) obtained by output values for *F*_1_ (0.32) and *F*_2_ (0.57) are 0.2 (L), 0.1 (M), 0.136 (L), and 0.358 (H), respectively, as presented in Figure 8 and Table 2. With these four values (0.2 (L), 0.1 (M), 0.136 (L), and 0.358 (H)), we can obtain four combinations of ((0.2 (L), 0.136 (L)), (0.2 (L), 0.358 (H)), (0.1 (M), 0.136 (L)), (0.1 (M), 0.358 (H)) as shown in Table 2.

Based on the fuzzy rules of Table 1 and assuming that we use min rule, we can obtain the four values as shown in Table 2. For example, 0.2 (VH) can be obtained with the second combination of (0.2 (L), 0.358 (H)). 0.1 (M) can be obtained with the fourth combination of (0.1 (M), 0.358 (H)).

**Figure 9:**
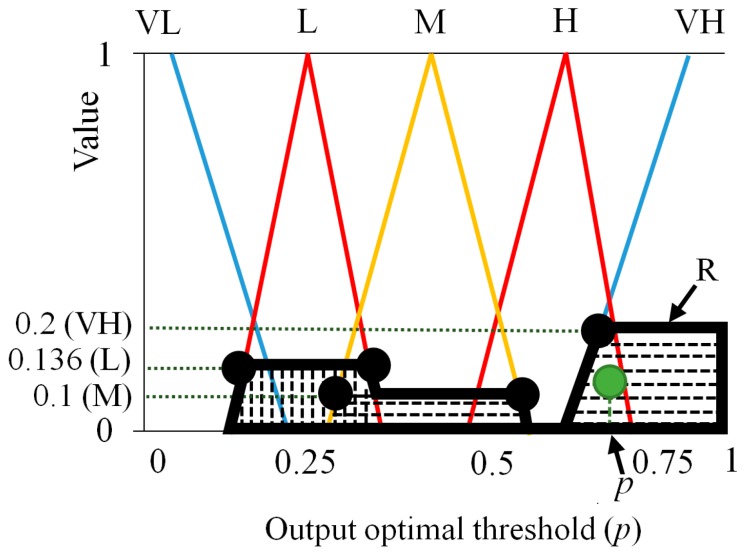
The first example for output optimal threshold based on the COG defuzzification method.

**Figure 10:**
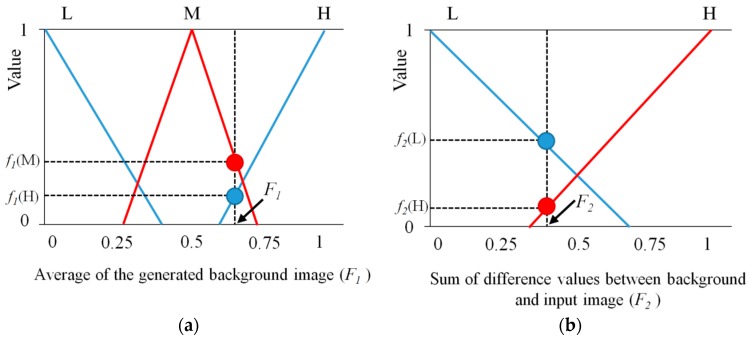
The second example for output of membership functions for fuzzy system: outputs by (**a**) *F*_1_ and (**b**) *F*_2_.

With these four values of (0.136 (L), 0.2 (VH), 0.1 (M), 0.1 (M)), we can define the region (R depicted by bold black lines of Figure 9) for obtaining the fuzzy output value. As shown in Figure 9, in the proposed method, center of gravity (COG) is used for the defuzzification method [44,45,46]. From the output membership function, which is illustrated in Figure 7 (as presented in Section 2.3.2), an output value called the output optimal threshold (p of the Equation (11)) is calculated as the gravity position of the region (R). As an example in the second instance, we assume that *f*_1_(M), *f*_1_(H), *f*_2_(L), and *f*_2_(H) obtained by output values for *F*_1_ (0.65) and *F*_2_ (0.4) are 0.25 (M), 0.125 (H), 0.394 (L), and 0.104 (H), respectively, as presented in Figure 10 and Table 3. With these four values (0.25 (M), 0.125 (H), 0.394 (L), and 0.104 (H)), we can obtain four combinations of ((0.25 (M), 0.394 (L)), (0.25 (M), 0.104 (H)), (0.125 (H), 0.394 (L)), (0.125 (H), 0.104 (H))) as shown in Table 3.

Based on the fuzzy rules of Table 1 and assuming that we use min rule, we can obtain the four values as shown in Table 3. For example, 0.25 (M) can be obtained with the first combination of (0.25 (M), 0.394 (L)). 0.125 (H) can be obtained with the third combination of (0.125 (H), 0.394 (L)).

**Figure 11:**
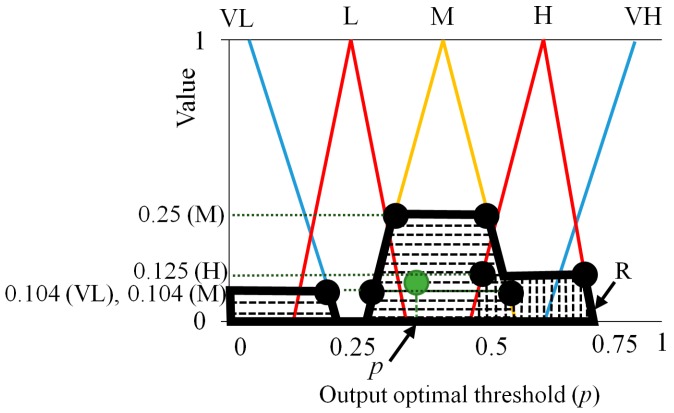
The second example for output optimal threshold based on the COG defuzzification method.

With these four values of (0.25 (M), 0.104 (M), 0.125 (H), 0.104 (VL)), we can define the region (R depicted by bold black lines of Figure 11) for obtaining the fuzzy output value. Based on the COG defuzzification method, the output optimal threshold (p of the Equation (11)) is calculated as the gravity position of the region (R), as illustrated in Figure 11.

#### 2.3.4. Generating a Difference Image

After extracting the optimal threshold by defuzzification, the threshold for human detection is calculated based on the Equation (11):

(11) $$\Theta_{th}=\;p\cdot \alpha +\beta$$

where: p is the optimal threshold from the fuzzy system and it has the range from 0 to 1; α and β are constants determined experimentally; and $\Theta_{th}$ is a threshold used to create the difference image presenting candidate human areas. The range of $\Theta_{th}$ is from β to α+β. The operation for generating a difference image is presented in Equation (12):

(12) $$D_{t}\left(i,j\right)=\{\begin{array}{c} \;1\;if\;\left|I_{k}\left(i,j\right)-B\left(i,j\right)\right|>\Theta_{th}\\ 0\;otherwise\; \end{array}$$

where: *B*(*i*, *j*) and *I_k_*(*i*, *j*) are the gray level values at the position (*i*, *j*) of a generated background and input image, respectively; *D_t_*(*i*, *j*) is a binarized image called a difference image in the proposed method; and *t* indicates the frame number of the input image in the sequence.

**Figure 12:**
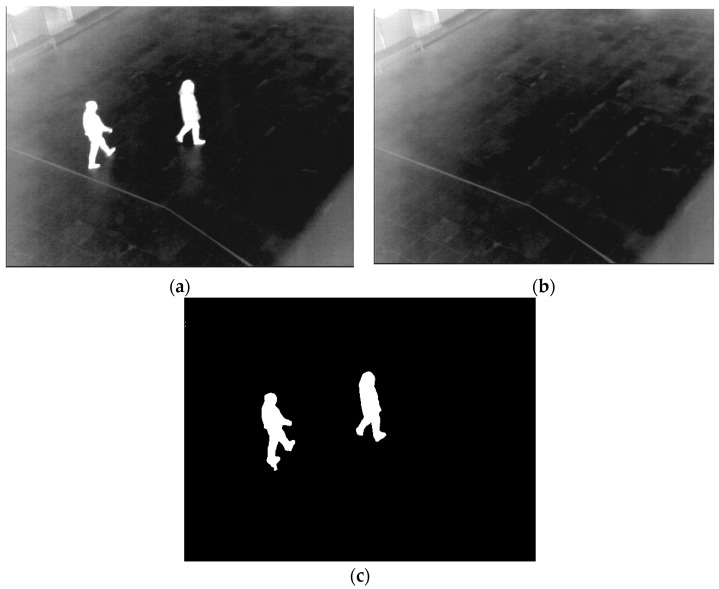
Example of a difference image: (**a**) input image; (**b**) background image; (**c**) difference image.

**Figure 13:**
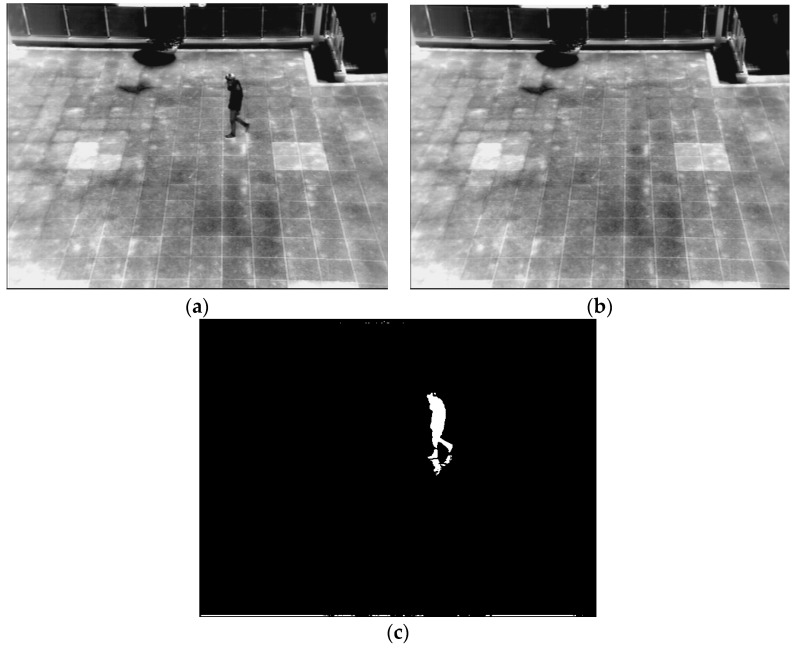
Example of a difference image: (**a**) input image; (**b**) background image; (**c**) difference image.

As illustrated in Figure 12 and Figure 13, the difference images presenting candidate human areas are correctly created by using the adaptive threshold. Even though the intensity of a human is darker than that of the background, the candidate area can be presented as shown in Figure 13.

### 2.4. Confirmation of Human Region

In the process of confirming a human area from a candidate area, several methods are used. First, morphological operation (dilation and erosion) and labeling are applied to the difference image to reduce incorrect human areas. Because the morphological operation including dilation and erosion on the candidate area can reduce the small-sized noises and combine the incorrectly separated regions [40]. Through the component labeling, the pixel positions of isolated candidate area can be located. Then, the pixel number of the candidate area can be counted [40]. Based on the pixel number, small or large areas (which is difficult to be regarded as human area) can be removed.

Then, separated small areas can be connected, and information concerning the candidate area can be more distinctive. However, when two or more people are connected, it is defined as one candidate region. Therefore, a histogram is used to separate the regions which include two or more humans region (see the details in Section 2.4.1).

#### 2.4.1. Vertical and Horizontal Separation of Candidate Region

**Figure 14:**
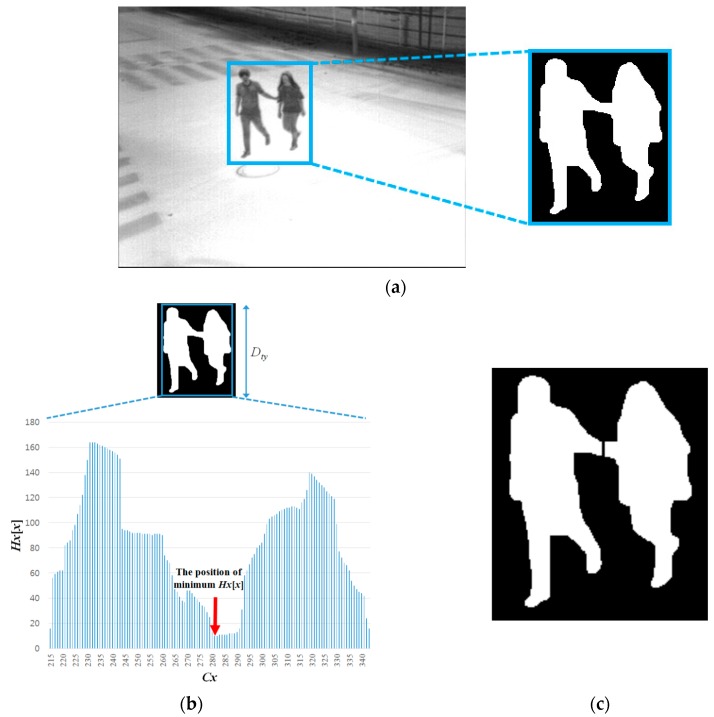
Separation of the candidate region within an input image based on the horizontal histogram: (**a**) input image and detected candidate region; (**b**) detected candidate region and its horizontal histogram; and (**c**) the division result of the candidate region.

**Figure 15:**
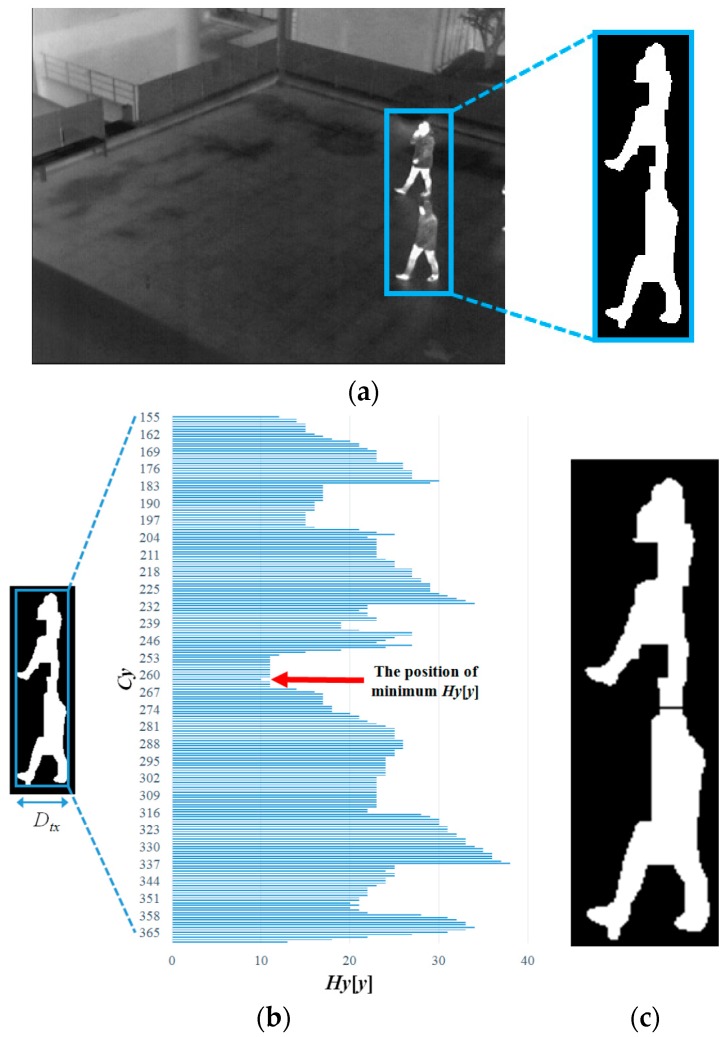
Separation of the candidate region within an input image based on the vertical histogram: (**a**) input image and detected candidate region; (**b**) detected candidate region and its vertical histogram; and (**c**) the division result of the candidate region.

If the condition (width, height, size, and ratio of height to width) of the candidate region is not satisfied with thresholds, the separation procedure is performed to the region. The position where the procedure of separation should be performed is determined by information in the histogram, as shown in Figure 14b and Figure 15b. If the minimum value of the histogram is lower than a parameter, separation is performed at the position and the candidate region is divided into two regions. Using Equations (13) and (14), the horizontal and vertical histograms are respectively presented [37,47,48]:

(13) $$Hx\left[x\right]=\overset{D_{ty}-1}{\underset{y=0}{\sum }}F\left(D_{t}\left(x,y\right)\right)$$

(14) $$Hy\left[y\right]=\overset{D_{tx}-1}{\underset{x=0}{\sum }}F\left(D_{t}\left(x,y\right)\right)$$

where: *D_t_*(*x*, *y*) is the pixel value at a location (*x*, *y*) of the candidate region, such that if *D_t_*(*x*, *y*) is true, *F*(·) is assigned to one and otherwise to zero; *D_ty_* and *D_tx_* are respectively the height and width of the candidate region, as in Figure 14b and Figure 15b, where *Cx* and *Cy* are the location *x* and *y* of the candidate region in the image, respectively; and *t* indicates the frame number of the input image in the sequence. 

**Figure 16:**
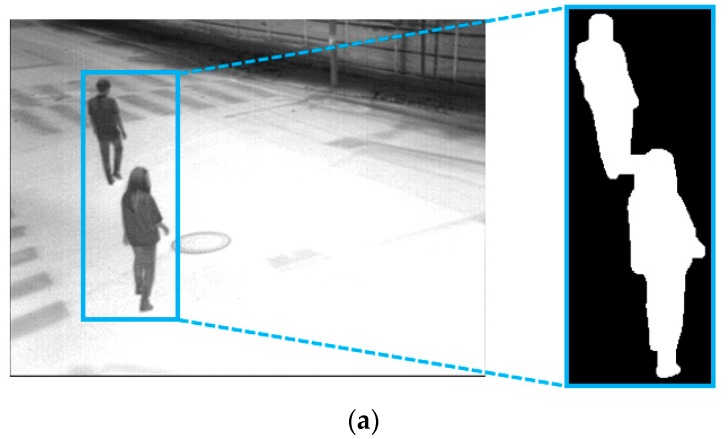

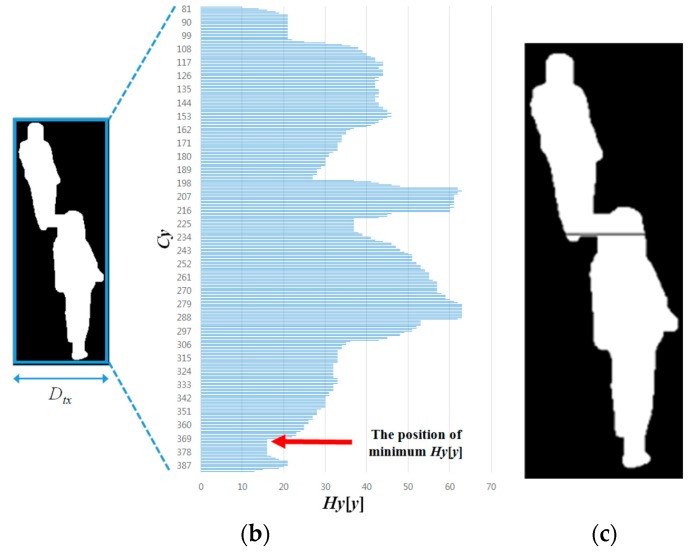
Separation of the candidate region within an input image based on the width, height, size and ratio: (**a**) input image and detected candidate region; (**b**) detected candidate region and its vertical histogram; and (**c**) the division result of the candidate region.

If the minimum value of *Hx*[*x*] or *Hy*[*y*], which is illustrated in Figure 14b or Figure 15b, is lower than the threshold, the candidate region is divided into two regions at the position, as illustrated in Figure 14c or Figure 15c. However, if two people are located closely in the diagonal direction or overlapped up and down, it is detected as one candidate region, which may not be separated by horizontal or vertical histogram information. As shown in Figure 16b, the minimum value of *Hy*[*y*] is higher than a threshold, and *Cy*, which is the position of the minimum value of *Hy*[*y*], is not located near the middle position of the candidate region, even though the region includes two people. In this case, if the conditions of the Equation (15) are satisfied, the candidate region is separated as two parts horizontally at the middle position of the candidate region as illustrated in Figure 16c, else the candidate region is not separated:

(15) $$\left(D_{ty}>thr_{1})\;\text{and}\;(D_{tx}<thr_{2})\;\text{and}\;(D_{ty}\times D_{tx}>thr_{3})\;\text{and}\;(D_{ty}/D_{tx}>thr_{4}\right)$$

where: *D_ty_* and *D_tx_* are respectively the height and width of the candidate region.

**Figure 17:**
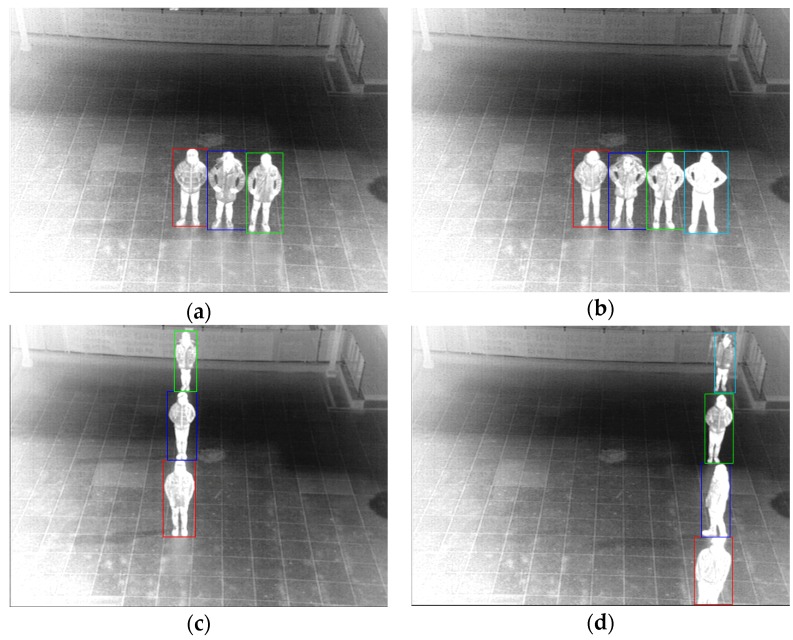
The examples of separation of one detected box into three (**a**,**c**) or four (**b**,**d**) ones by our method.

In order to consider the cases of three or more people in the similar place with occlusion, if the horizontal width of the detected box is larger than threshold, our algorithm checks whether there exist two or more than two values of *Hx*[*x*] of the Equation (13) which are lower than the threshold. If so, the candidate region is horizontally divided into three or more than three regions at the positions of the values of *Hx*[*x*]. Same method is applied based on *Hy*[*y*] of the Equation (14) for the vertical division of the detected box. Figure 17 shows the examples of separation of one detected box into three or four ones by our method.

Our novel ideas in this Section are to segment two people with occlusion in diagonal direction (Figure 16) and handle with the cases of three or more people in the similar place with occlusion (Figure 17). All the parameters used in the method of dividing the detected box were experimentally determined with the images (which were not used for all the experiments of performance measurements shown in Section 3) in advance. With these images, the ground-truth areas of human were manually depicted. In addition, according to various parameters, the human areas could be automatically detected. With the ground-truth and automatically detected areas, we can calculate the PPV, Sensitivity, and F1-score of the Equations (17)–(19). Optimal parameters were determined, with which the highest PPV, Sensitivity, and F-score of human detection were obtained. In addition, the same parameters were used for all the experiments in Section 3.

#### 2.4.2. Confirmation of Human Area Based on Camera Viewing Direction

**Figure 18:**
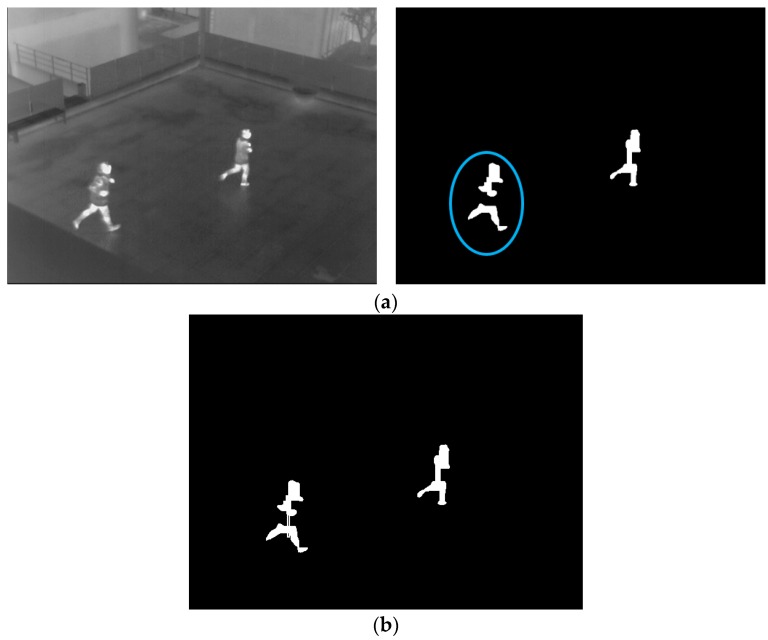
Separation of the candidate region within an input image: (**a**) input image and candidate human regions; (**b**) result of connecting separated regions.

To remove the incorrect human areas, component labeling [40] and size filtering are applied to the binarized image. If the size of the candidate region is too small or large, the region is determined to be an incorrect human area and removed. Then, candidates for human areas remain, but some parts are separated and located closely as shown in Figure 18a (blue ellipse). To define the regions as one object, a procedure, which connects the regions, is applied to the binarized image based on the size, the horizontal and diagonal distances between center positions of the two objects, and the camera viewing direction.

**Figure 19:**
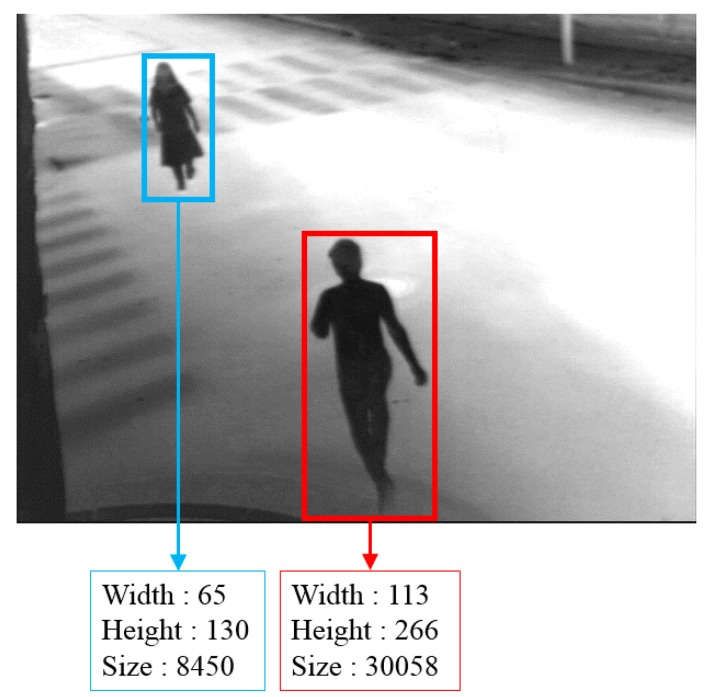
Example of different sizes of human areas because of camera viewing direction.

If the size of a region and distances are satisfied with the conditions, these two regions are connected and are defined as one region, as shown in Figure 18b. In general, the size of the human captured at the upper area of the image is smaller than that of the human located in the bottom area, as shown in Figure 19 due to the Z distance between the object and camera. Therefore, if there are small parts located in the upper area of the image, the procedure for connection is not performed. On the other hand, if there are small parts located in lower are of the image, the procedure is performed. This procedure is implemented iteratively to all detected candidate regions.

**Figure 20:**
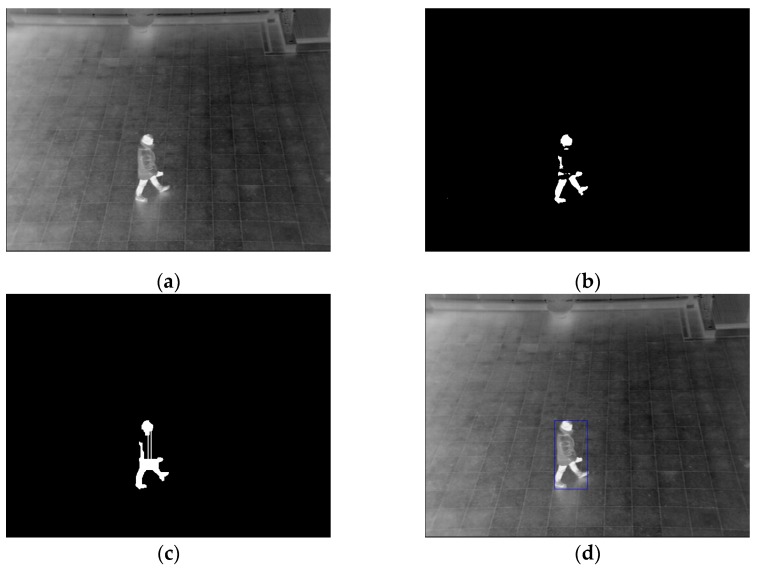
Example of procedures for detecting human regions: (**a**) input image; (**b**) binarized image by background subtraction; (**c**) result of connecting separated candidate regions; and (**d**) Final result of detected human area.

Figure 20 shows the example of human detection by this procedure. After the blobs are merged, the method is not applied again to the obtained blobs, and final detected area of human is obtained as shown in Figure 20d. Our algorithm can handle with the cases that more than two (multiple) blobs should be merged.

## 3. Experimental Results

### 3.1. Dataset Description

For the experiments, 15 thermal databases collected by us were utilized. The databases for the experiments are captured by a FLIR Tau 2 (in the wavelength range of 7.5–13.5 μm) thermal camera [49] equipped with a 19 mm lens. In our research, we use the assumption that the camera position is fixed. Our camera is tilted and set at the height of about 6~8 m from the ground. The distance between the camera and object is approximately 21~27 m. The fields of view for the camera in the horizontal and vertical directions are 32° and 26°, respectively. These specifications of height and distance have been widely used in conventional surveillance camera system, and we collected our databases based on these specifications. The size of images is 640 × 480 pixels of 8 bits. Each database contains between 905 and 5599 frames. The total number of images for all databases is 45,546. The sizes of humans in width and height range from 28 to 117 and from 57 to 265 pixels, respectively. In our research, all the parameters of our system were set with the dataset of 985 images. This dataset is different from the 15 databases of 45,546 images (Table 4) which are used for testing our system. For validation of applicability of the proposed method to the various databases, we captured databases at different temperatures and conditions, such as different times, weather conditions, views, and places. The frames in the databases include winter and summer temperatures between −6 °C and 33 °C.

In general, the human region is brighter than that of the background in frames captured by a thermal camera. However, if the temperature of the ground is too high, the brightness of human region is darker than that of the background in the frames. The reason for this is that the thermal camera performs automatically to create an 8 bit image, which is presented in the range of 0 to 255 pixel value.

**Figure 21:**
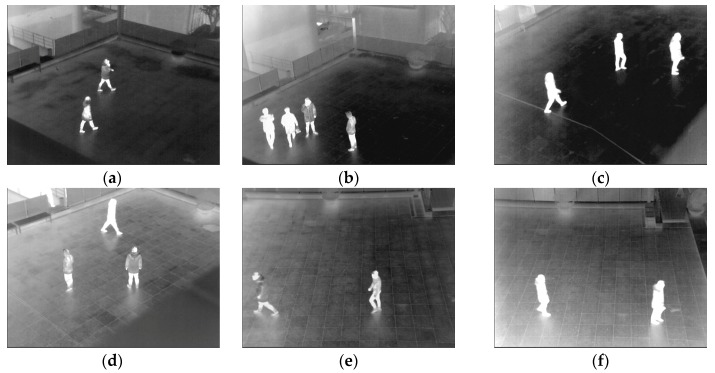

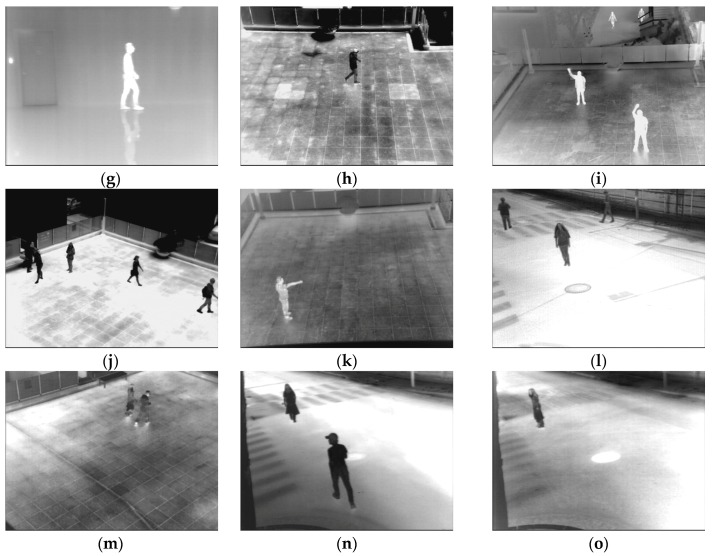
Examples of databases: (**a**) database I; (**b**) database II; (**c**) database III; (**d**) database IV; (**e**) database V; (**f**) database VI; (**g**) database VII; (**h**) database VIII; (**i**) database IX; (**j**) database X; (**k**) database XI; (**l**) database XII; (**m**) database XIII; (**n**) database XIV; and (**o**) database XV.

Databases I–VI, VIII–XI, and XIII are obtained by a thermal camera placed 6 m above the ground level, with the Z distance from object to camera being about 25 m in most cases. Database VII only includes frames of an indoor environment. The database is captured from a camera placed 2 m above the ground level, with the Z distance from the object to the camera being 12 m. Databases XII, XIV, and XV are obtained by a camera placed 4.5 m above the ground level, with the Z distance from the object to the camera being approximately 18 to 35 m. There are various behaviors of people in frames, such as walking, running, standing, sitting, waving, kicking, and punching. Motionless people, including people standing or sitting, are presented in databases I–VIII and XIV. A detailed description for each database is presented in Table 4, and the examples of the databases are shown in Figure 21.

### 3.2. Results of Generating Background Model

**Figure 22:**
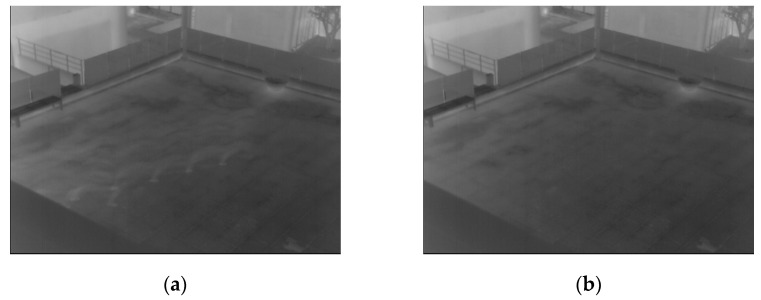
Comparisons of preliminary background images with database I. The left figure (**a**) is by [33,34,35] and right figure (**b**) is by the proposed method, respectively.

**Figure 23:**
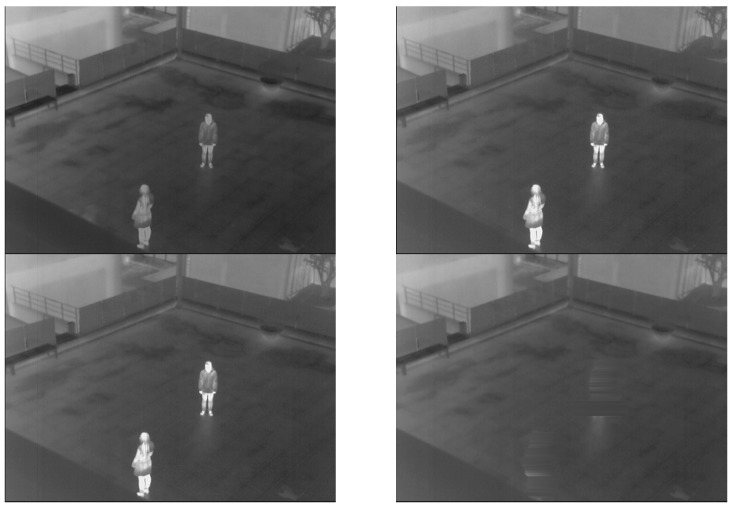
Comparisons of created background images with database I. The left-upper, right-upper, left-lower and right-lower figures are by [24,27,28,29,30,31,33,34,35] and the proposed method, respectively.

**Figure 24:**
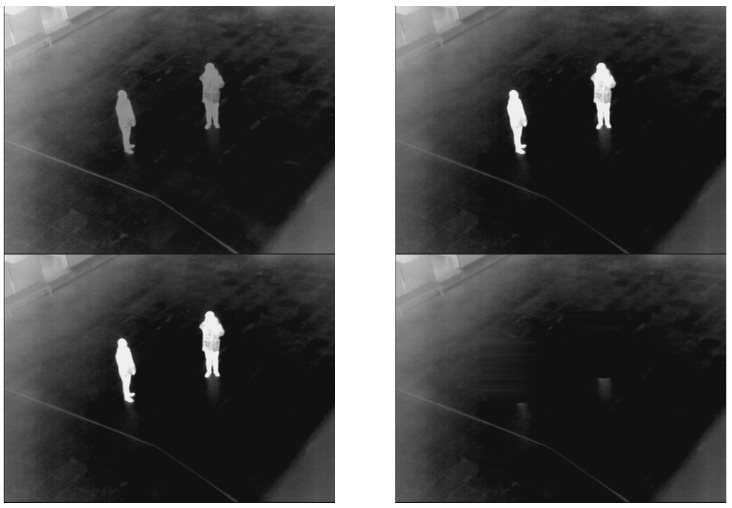
Comparisons of created background images with database III. The left-upper, right-upper, left-lower and right-lower figures are by [24,27,28,29,30,31,33,34,35] and the proposed method, respectively.

As the first experiment, a background image from the proposed method is compared to those from other methods as shown in Figure 22, Figure 23 and Figure 24. Most previous research created a background image by using a simple averaging operation with multiple frames. However, some ghost shadows can exist, as illustrated in Figure 22. Those ghost shadows are from a high-level intensity of humans included in the frames. By using the median pixel values, more correct background images can be created by our method.

As shown in Figure 23, if there are motionless people in all frames, human areas are shown in background images by averaging methods [33,34,35]. To overcome this drawback, previous research [24] utilized the averaging of two different sequences to create the correct background image. However, if there is a tree or vehicle in a sequence, there is a brightness change in the created image compared to the input images. This brightness change can influence the generation of erroneous detection results by background subtraction. Therefore, maintaining the brightness of a generated background image compared to the input image is important in the use of the background subtraction technique. 

In other research [27,28,29,30,31], statistical modeling was used by calculating weighted means and variances of the sampled values to create a background image. All of these methods have problems concerned with the inclusion of humans in the generated background image, as shown in Figure 23. On the other hand, humans are completely removed in a background images generated by our method. Additional examples of comparison are presented in Figure 24.

### 3.3. Detection Results

**Figure 25:**
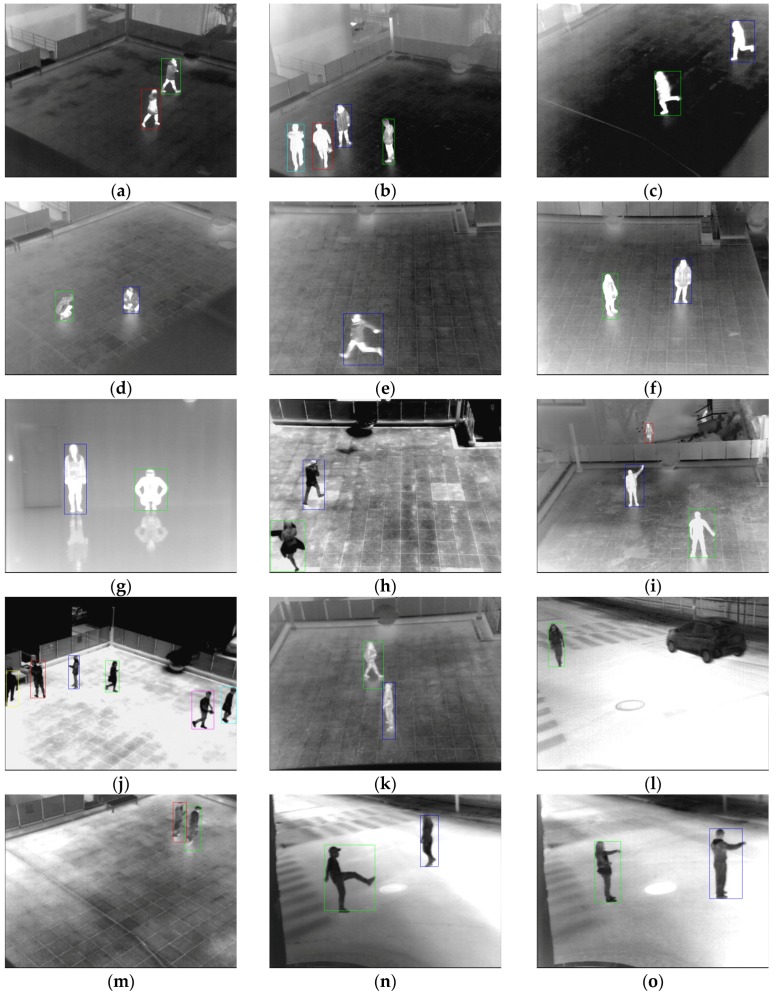
Detection results with database (**I**–**XV**). Results of images in: (**a**) Database I; (**b**) Database II; (**c**) Database III; (**d**) Database IV; (**e**) Database V; (**f**) Database VI; (**g**) Database VII; (**h**) Database VIII; (**i**) Database IX; (**j**) Database X; (**k**) Database XI; (**l**) Database XII; (**m**) Database XIII; (**n**) Database XIV; and (**o**) Database XV.

In Figure 25, the detection results of the proposed method are presented. The square box indicates the detected region of a human. Despite the fact that there are humans located closely (and with a little overlap) (Figure 25a,b,j,k,m), various types of human areas, such as human areas darker than the background (Figure 25h,j,l–o), vehicles (Figure 25l), similar intensities between humans and the background (Figure 25d,k,m), and various types of human behavior, such as walking, running, sitting, standing, waving, punching, and kicking (Figure 25a–o), are detected correctly. As shown in Figure 25, complex scene does not affect the human detection because the image by thermal camera is not changed according to the complexity of scene but the temperature of the scene.

Next, for quantitative evaluation of the detection accuracies by the proposed method, we manually set square boxes surrounding human areas as ground truth regions. The detection results were evaluated with true or false positives by measuring the overlap area of a ground truth and a bounded box based on the PASCAL measure [50,51,52]. If the overlap area $O_{dg}$ from a detected bounding box *B_db_* and a ground truth box *B_gt_* exceeded threshold, we counted the result as a true positive case, which means a correct detection. The overlap is calculated using Equation (16):

(16) $$O_{dg}=\;\frac{area(B_{db}\cap B_{gt})}{area(B_{db}\cup B_{gt})}$$

where: *B_db_* ∩ *B_gt_* presents the intersection of the detected and the ground truth bounding boxes; and *B_db_* ∪ *B_gt_* is their union [50,51,52]. Based on Equation (16), the number of true positive (TP) and false positive (FP) are counted. The positive and negative samples represent the human and background areas, respectively. Therefore, TPs are the correct detection results and FPs are the incorrect cases. False negative (FN) are the number of humans not detected using the proposed method. That is, the total number of TP and FN is the total number of human regions in all the images.

Based on this, the positive predictive value (PPV) (precision) and sensitivity (recall) are obtained, as indicated in Equations (17) and (18) [15,53]. In these equations, the number of TP, FP, and FN cases are represented as #TP, #FP, and #FN, respectively. To present a single value for accuracy, the F1-score is obtained by PPV and sensitivity [54]. Therefore, a higher value for the F1-score means a higher accuracy of human detection. The operation for obtaining the F1-score is presented in Equation (19):

(17) $$\text{PPV}=\;\frac{\#\text{TP}}{\#\text{TP}+\#\text{FP}}$$

(18) $$\text{Sensitivity}=\;\frac{\#\text{TP}}{\#\text{TP}+\#\text{FN}}$$

(19) $$F1-\text{Score}=2\times \frac{Sensitivity\times PPV}{Sensitivity+PPV}$$

As indicated in Table 5, the detection accuracy of the proposed method with fifteen databases is presented. The PPV, sensitivity, and F1-score are 95.01%, 96.93%, and 95.96%, respectively. Database III, captured at early morning and at 0 °C, shows the best results. The contrast between humans and backgrounds in frames is very clear. Therefore, the detection accuracies obtained with the database III are higher than other results. On the other hand, database XII captured on a hot summer day shows worse results. The temperature of the scene rises above 27 °C, and the average temperature of that day was 23 °C. Because of the temperature (around 25 °C), humans appear to be much darker than the background. In addition, there are much darker areas than other regions, which are similar to human regions, even though the area is not a human area but rather a background area. This is due to the temperature of buildings and roads, which received heat. Moreover, there are some occluded humans in frames in the database. Because of these factors, the F1-score of the database is 80.33%, which is lower than other results, but still satisfactory. If the temperature at that time is above 27 °C and average temperature is above 25 °C, the area of the human is shown as being much darker than other areas. Therefore, the results from the databases XIII–XV are higher than the results from database XII.

In Table 6, the detection accuracy categorized by human behaviors is presented. The sitting case shows the best results. This means that the generated background image is created correctly for the background subtraction method. The walking case presents comparatively worse results. This is because there are occlusions in several frames. The PPV, sensitivity, and F1-score shows 90.46%, 93.78%, and 92.09%, which are lower than other results, but these results remain acceptable. Based on Table 5 and Table 6, we can conclude that the proposed method can detect humans correctly given various environmental conditions and behaviors of humans.

**Figure 26:**
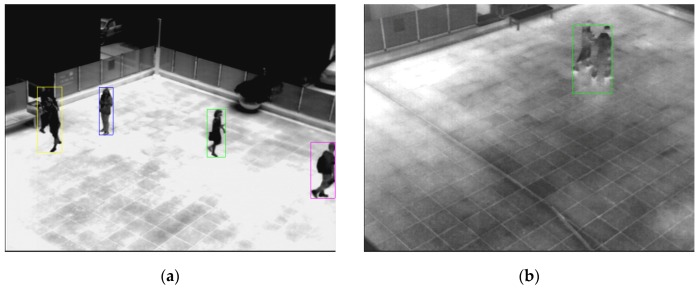
Detection error cases: (**a**) result of the proposed method with database X; (**b**) result of the proposed method with database XIII.

In Figure 26, we show the detection error cases by the proposed method. As shown in Figure 26a, there are two people in the middle-left area of the image. However, because of the occlusion of the two humans, error cases occur, indicated by a drawn yellow square box. Further, there are, as shown in Figure 26b, two people in the upper-right area of the image. However, one candidate region is detected, with a green square box.

In next experiments, we performed the comparisons with existing methods [24,32,37]. Same databases used in Table 5 and Table 6 were used for comparisons. The comparative results of human detections are shown in Table 7 and Table 8.

In addition, we performed the additional comparisons with existing methods [24,32,37] on other database (OSU thermal pedestrian database of object tracking and classification beyond visible spectrum (OTCBVS) benchmark dataset [32,55]). This database has been widely used as an open database for measuring the performance of object detection with the images by thermal camera. It includes ten categorized sequences of thermal images which were collected in different weather condition and different time. The comparative results of human detections are shown in Table 9. As shown in the Table 7, Table 8 and Table 9, we can confirm that our method outperforms the previous methods [24,32,37] with both our database and OTCBVS database.

As explained in Figure 1 and Section 2.4.2, our method removes the incorrect human areas based on the size and ratio information (the ratio of height to width) of the detected box. Because the size and the ratio (of height to width) of the detected dog area are comparatively smaller than those of human area, respectively, the detected box of dog can be removed from the candidates of detected human region by our method. However, other animals whose size, ratio, and temperature are similar to those of human can be detected as incorrect human area.

## 4. Conclusions

In this research, we presented a new method of detecting humans in thermal images based on the generation of a background image and fuzzy system under various environmental conditions. A correct background image was generated using a median image and through erasing methods of human areas. A difference image was obtained using a fuzzy system, which is used to determine thresholds adaptively. Human candidate regions were divided based on histogram information. The regions were redefined based on the size and the ratio of humans, with camera view being taken into consideration. Based on the redefined candidate region, human areas were detected. Through experiments in various environments, we proved the effectiveness of the proposed system. In future work, we will study solutions for solving the problems caused by occlusion. In addition, we would expand the research in human behavior classification.

## Figures and Tables

**Table 1:** Fuzzy rules based on the characteristics of the background and input images.

Input 1 (*F*_1_)	Input 2 (*F*_2_)	Output (*p)*
L	L	L
L	H	VH
M	L	M
M	H	M
H	L	H
H	H	VL

**Table 2:** The first example for fuzzy rules and min rule based on the characteristics of the background and input images.

*f*_1_(·)	*f*_2_(·)	Value
0.2 (L)	0.136 (L)	0.136 (L)
0.2 (L)	0.358 (H)	0.2 (VH)
0.1 (M)	0.136 (L)	0.1 (M)
0.1 (M)	0.358 (H)	0.1 (M)

**Table 3:** The second example for fuzzy rules and min rule based on the characteristics of the background and input images.

*f*_1_(·)	*f*_2_(·)	Value
0.25 (M)	0.394 (L)	0.25 (M)
0.25 (M)	0.104 (H)	0.104 (M)
0.125 (H)	0.394 (L)	0.125 (H)
0.125 (H)	0.104 (H)	0.104 (VL)

**Table 4:** Descriptions of fifteen databases.

Database	Condition	Detail Description
I (see in Figure 21a)	2 °C, morning, average −1 °C during the day, snowy, wind 3.6 mph	- The behaviors of the humans include walking, running, standing, and sitting. - The sequence was captured during a little snowfall. - The intensity of the human is influenced by material of clothes.
II (see in Figure 21b)	−2 °C, night, average −3 °C during the day, wind 2.4 mph	- The behaviors of the humans include walking, running, standing, and sitting. - Three or four people appear together in several frames. - Example of this database is presented in Figure 21b.
III (see in Figure 21c)	−1 °C, morning, average 3 °C during the day, sunny after rainy at dawn time, wind 4.0 mph	- The behaviors of the humans include walking, running, standing, and sitting. - The brightness of the human is very different compared to that of the background. - The pixel value of the human is much higher than that of the background.
IV (see in Figure 21d)	−6 °C, night, average −3 °C during the day, sunny after rainy at dawn time, wind 4.0 mph	- The behaviors of the humans include walking, running, standing, and sitting. - The intensity of the human is variously affected by temperature. - If a person just appears from a building (indoors), the brightness of the person is much greater than other objects. The day when the database was captured was too cold.
V (see in Figure 21e)	−2 °C, night, average −2 °C during the day, sunny, wind 4.9 mph	- The behaviors of the humans include walking, running, standing, and sitting. - There is a person wearing thick clothes. Therefore, the brightness of human is similar to the background because the intensity of image captured by infrared camera depends on the emission of heat.
VI (see in Figure 21f)	−1 °C, morning, average 2 °C during the day, sunny, wind 2.5 mph	- The behaviors of the humans include walking, running, standing, and sitting. - The Halo effect is shown below the regions of humans. It is shown distinctive to the background. - The brightness of the humans is much higher than that of background.
VII (see in Figure 21g)	22 °C, indoor, average −12 °C during the day outside, no wind	- The behaviors of the humans include walking, running, standing, and sitting. - The brightness of an image captured indoors is brighter than that of an image captured outside. - The reflected region is located under the human region. The size of the region is same with the human. It is influenced by the material of the floor.
VIII (see in Figure 21h)	26 °C, afternoon, average 21 °C during the day, sunny, wind 1 mph	- The behaviors of the humans include walking, sitting, and waving. - The intensity of the humans is much lower than the background. The intensity of some background regions is also similar to that of human.
IX (see in Figure 21i)	14 °C, morning, average −18 °C during the day, sunny, wind 2.4 mph	- The behavior of humans is waving. - There are two or four people in the sequence. Their sizes are very different. - The intensity of the humans is higher than that of background. There is also a watering ground.
X (see in Figure 21j)	28 °C, afternoon, average −23 °C during the day, sunny, wind 5 mph	- The behavior of humans is walking. - The sequence is captured during a hot day. - The intensity of the image is influenced by the camera module system. Therefore, the brightness of humans is much darker than that of the background. - There are some occluded people that can be a cause of difficulty for detection of the proposed system.
XI (see in Figure 21k)	18 °C, night, average 19 °C during the day, sunny after rainfall during the daytime, wind 2 mph	- The behaviors of the human include kicking and punching. - The person that appeared in the sequence is wearing short sleeves. - The intensity of the human is a little higher than that of the background.
XII (see in Figure 21l)	27 °C, afternoon, average 23 °C during the day, sunny, wind 4.3 mph	- The behavior of the humans is walking. - There is region whose brightness is very similar to humans. Intensity of humans is reflected because of the fences. Not only the size but also intensity of the reflection is very similar to those of humans. - The sequence is captured during a hot day. - The intensity of the image is influenced by the camera module system. There is a slight brightness change during recording because of a large vehicle.
XIII (see in Figure 21m)	27 °C, night, average 29 °C during the day, sunny after rainfall during morning, wind 2.4 mph	- The behaviors of the humans include walking, waving, and punching. - The intensity of the human is similar to that of the background. The detection result of the proposed method is affected by the little contrast between humans and the background.
XIV (see in Figure 21n)	33 °C, afternoon, average 29 °C during the day, sunny, wind 3.5 mph	- The behaviors of the humans include walking, running, standing, punching, and kicking. - The sequence is captured during a heat wave. - The humans that appeared in the sequence are wearing short sleeves. - The brightness of the humans is darker than that of the background. There is a region whose brightness is very similar to the background. There are two crosswalks whose intensity is a little darker than the surrounding region. - There is a slight brightness change during the recording because of a large vehicle.
XV (see in Figure 21o)	30 °C, night, average 29 °C during the day, sunny, wind 2.5 mph	- The behaviors of the human include walking, waving, kicking, and punching. - The intensity of the human is much darker than the background. A human is shown relevant to the background region. - There is a round iron piece in the middle of the images. There is a region whose brightness is very similar to the background. There are two crosswalks whose intensity is a little darker than the surrounding region.

**Table 5:** Results of human detection by the proposed method with our database.

Database No.	#Frames	#People	#TP	#FP	Sensitivity	PPV	F1-Score
I	2609	3928	3905	48	0.9941	0.9879	0.9910
II	2747	4543	4536	135	0.9985	0.9711	0.9846
III	3151	5434	5433	60	0.9998	0.9891	0.9944
IV	3099	4461	4368	101	0.9792	0.9774	0.9783
V	4630	5891	5705	113	0.9684	0.9806	0.9745
VI	3427	3820	3820	70	1	0.9820	0.9909
VII	3330	3098	3046	14	0.9832	0.9954	0.9893
VIII	1316	1611	1505	58	0.9342	0.9629	0.9483
IX	905	2230	1818	0	0.8152	1	0.8982
X	1846	3400	3056	112	0.8988	0.9646	0.9306
XI	5599	6046	5963	162	0.9863	0.9736	0.9799
XII	2913	4399	3407	676	0.7745	0.8344	0.8033
XIII	3588	4666	4047	33	0.8673	0.9919	0.9255
XIV	5104	7232	7036	158	0.9729	0.9780	0.9755
XV	1283	1924	1913	148	0.9942	0.9282	0.9601
Total	45,546	62,683	59,558	1888	0.9501	0.9693	0.9596

**Table 6:** Results of human detection categorized by human behaviors with our database.

Behavior	#Frames	#People	#TP	#FP	Sensitivity	PPV	F1-Score
Walking	17,380	22,315	20,186	1340	0.9046	0.9378	0.9209
Running	6274	3864	3776	153	0.9772	0.9611	0.9536
Standing	5498	10,430	10,356	67	0.9929	0.9936	0.9932
Sitting	6179	11,417	11,364	3	0.9954	0.9997	0.9975
Waving	1975	3611	3181	0	0.8809	1	0.9367
Punching	3932	5434	5117	96	0.9417	0.9816	0.9612
Kicking	4308	5612	5578	229	0.9939	0.9606	0.9770

**Table 7:** Comparative results of human detection by the proposed method and previous ones [24,32,37] with our database.

DB No.	Sensitivity					PPV				F1-Score		
	Ours	Previous Method			Ours	Previous Method			Ours	Previous Method		
		[24]	[32]	[37]		[24]	[32]	[37]		[24]	[32]	[37]
I	0.9941	0.9514	0.9351	0.9832	0.9879	0.9544	0.8713	0.9621	0.9910	0.9529	0.9021	0.9725
II	0.9985	0.9595	0.9406	0.9885	0.9711	0.9462	0.8623	0.9539	0.9846	0.9528	0.8998	0.9709
III	0.9998	0.9522	0.9366	0.9763	0.9891	0.9515	0.8711	0.9597	0.9944	0.9519	0.9027	0.9679
IV	0.9792	0.9386	0.9219	0.9698	0.9774	0.9497	0.8698	0.9678	0.9783	0.9441	0.8951	0.9688
V	0.9684	0.9257	0.9085	0.9559	0.9806	0.9605	0.8792	0.9681	0.9745	0.9428	0.8936	0.9620
VI	1	0.9601	0.9441	0.9913	0.9820	0.9525	0.8712	0.9696	0.9909	0.9563	0.9062	0.9803
VII	0.9832	0.9432	0.9231	0.9714	0.9954	0.9644	0.8823	0.9713	0.9893	0.9537	0.9022	0.9714
VIII	0.9342	0.9001	0.8792	0.9278	0.9629	0.9399	0.8581	0.9473	0.9483	0.9196	0.8685	0.9374
IX	0.8152	0.7653	0.7554	0.8049	1	0.9731	0.8923	0.9815	0.8982	0.8568	0.8182	0.8845
X	0.8988	0.8509	0.8325	0.8811	0.9646	0.9327	0.8498	0.9409	0.9306	0.8899	0.8411	0.9100
XI	0.9863	0.9414	0.9225	0.9709	0.9736	0.9497	0.8612	0.9573	0.9799	0.9455	0.8908	0.9641
XII	0.7745	0.7278	0.7105	0.7592	0.8344	0.8121	0.7193	0.8199	0.8033	0.7676	0.7149	0.7884
XIII	0.8673	0.8198	0.8019	0.8509	0.9919	0.9623	0.8802	0.9793	0.9255	0.8854	0.8392	0.9106
XIV	0.9729	0.9309	0.9113	0.9599	0.9780	0.9431	0.8621	0.9518	0.9755	0.9370	0.8860	0.9558
XV	0.9942	0.9502	0.9351	0.9825	0.9282	0.8976	0.8064	0.9056	0.9601	0.9232	0.8660	0.9423
Avg	0.9501	0.9064	0.8896	0.9376	0.9693	0.9409	0.8573	0.9505	0.9596	0.9234	0.8731	0.9437

**Table 8:** Comparative results of human detection categorized by human behaviors by the proposed method and previous ones [24,32,37] with our database. (Behav.: Behavior, W: Walking, R: Running, St: Standing, Si: Sitting, Wav: Waving, P: Punching, K: Kicking).

Behav.	Sensitivity					PPV				F1-Score		
	Ours	Previous Method			Ours	Previous Method			Ours	Previous Method		
		[24]	[32]	[37]		[24]	[32]	[37]		[24]	[32]	[37]
W	0.9046	0.8612	0.8434	0.8923	0.9378	0.9084	0.8269	0.9175	0.9209	0.8842	0.8351	0.9047
R	0.9772	0.9331	0.9193	0.9629	0.9611	0.9034	0.8203	0.9103	0.9536	0.9180	0.8670	0.9359
St	0.9929	0.9474	0.9295	0.9735	0.9936	0.9652	0.8812	0.9713	0.9932	0.9562	0.9047	0.9724
Si	0.9954	0.9523	0.9378	0.9821	0.9997	0.9703	0.8903	0.9785	0.9975	0.9612	0.9134	0.9803
Wav	0.8809	0.8371	0.8198	0.8656	1	0.9702	0.8913	0.9798	0.9367	0.8987	0.8541	0.9192
P	0.9417	0.9005	0.8837	0.9334	0.9816	0.9527	0.8702	0.9605	0.9612	0.9259	0.8769	0.9468
K	0.9939	0.9492	0.9302	0.9793	0.9606	0.9311	0.8525	0.9556	0.9770	0.9401	0.8897	0.9673

**Table 9:** Comparative results of human detection by the proposed method and previous ones [24,32,37] with OTCBVS database. (Seq. No.: Sequence Number).

Seq. No.	Sensitivity					PPV				F1-Score		
	Ours	Previous Method			Ours	Previous Method			Ours	Previous Method		
		[24]	[32]	[37]		[24]	[32]	[37]		[24]	[32]	[37]
1	1	1	0.97	1	1	1	1	1	1	1	0.9848	1
2	1	0.99	0.94	1	1	1	1	1	1	0.9949	0.9691	1
3	0.99	0.99	1	0.98	0.99	0.98	0.99	0.99	0.99	0.9850	0.9950	0.9850
4	1	1	0.98	1	0.99	1	0.99	0.97	0.9950	1	0.9850	0.9848
5	1	1	0.89	1	1	1	1	1	1	1	0.9418	1
6	0.99	1	0.96	0.98	1	1	1	1	0.9950	1	0.9796	0.9899
7	1	1	0.98	1	1	1	1	1	1	1	0.9899	1
8	1	1	0.76	1	1	0.99	0.99	1	1	0.9950	0.8599	1
9	1	1	1	1	1	1	1	1	1	1	1	1
10	1	0.97	0.98	1	1	0.97	0.97	1	1	0.97	0.9750	1
Avg	0.9980	0.9949	0.9459	0.9959	0.9980	0.9939	0.9936	0.9959	0.9980	0.9945	0.9680	0.9960
